# The Metabolic Signature of Cardiorespiratory Fitness

**DOI:** 10.1111/sms.70034

**Published:** 2025-03-12

**Authors:** Julia Bork, Marcello R. P. Markus, Ralf Ewert, Matthias Nauck, Christian Templin, Henry Völzke, Gabi Kastenmüller, Anna Artati, Jerzy Adamski, Marcus Dörr, Nele Friedrich, Martin Bahls

**Affiliations:** ^1^ Department of Internal Medicine B University Medicine Greifswald Greifswald Germany; ^2^ German Centre for Cardiovascular Research (DZHK) Partner Site Greifswald Greifswald Germany; ^3^ German Center for Diabetes Research (DZD) Partner Site Greifswald Greifswald Germany; ^4^ Institute of Clinical Chemistry and Laboratory Medicine University Medicine Greifswald Greifswald Germany; ^5^ Institute for Community Medicine, SHIP‐KEF University Medicine Greifswald Greifswald Germany; ^6^ Institute of Bioinformatics and Systems Biology Helmholtz Zentrum München Neuherberg Germany; ^7^ Metabolomics and Proteomics Core Helmholtz Center Munich Neuherberg Germany; ^8^ Institute of Experimental Genetics, Helmholtz Zentrum München German Research Center for Environmental Health Neuherberg Germany; ^9^ Department of Biochemistry, Yong Loo Lin School of Medicine National University of Singapore Singapore Singapore; ^10^ Institute of Biochemistry, Faculty of Medicine University of Ljubljana Ljubljana Slovenia

**Keywords:** cardiorespiratory fitness, epidemiology, exercise testing, metabolomics

## Abstract

High cardiorespiratory fitness (CRF) is associated with better overall health. This study aimed to find a metabolic signature associated with CRF to identify health‐promoting effects. CRF based on cardiopulmonary exercise testing, targeted and untargeted metabolomics approaches based on mass spectrometry, and clinical data from two independent cohorts of the Study of Health in Pomerania (SHIP) were used. Sex‐stratified linear regression models were adjusted for age, smoking, and height to relate CRF with individual metabolites. A total of 132 (SHIP‐START‐2: 483 men with a median age of 58 years and 450 women with a median age of 56 years) and 118 (SHIP‐TREND‐0: 341 men and 371 women both with a median age of 51 years) metabolites were associated with CRF. Lipids showed bidirectional relations to CRF independent of sex. Specific subsets of sphingomyelins were positively related to CRF in men (SM (OH) C14:1, SM(OH)C22:2 SM C16:0, SM C20:2 SM(OH)C24:1) and inversely in women (SM C16:1, SM C18:0, SM C18:1). Metabolites involved in energy production (citrate and succinylcarnitine) were only associated with CRF in men. In women, xenobiotics (hippurate, stachydrine) were related to CRF. The sex‐specific metabolic signature of CRF is influenced by sphingomyelins, energy substrates, and xenobiotics. The greater effect estimates seen in women may emphasize the important role of CRF in maintaining metabolic health. Future research should explore how this profile changes with different types of exercise interventions or diseases in diverse populations and how these metabolites could be implemented in primary prevention settings.

**Abbreviations**
BCAA: branched chain amino acids
BMI: Body Mass Index
CKD‐EPI: Chronic Kidney Disease—Epidemiology Collaboration
CRF: cardiorespiratory fitness
IR: insulin resistance
SHIP: Study of Health in Pomerania
T2DM: Type 2 Diabetes mellitus
VO_2_peak: peak oxygen uptake

## 1 Introduction

Cardiorespiratory fitness (CRF), which may be measured using VO_2_peak (mL/min/kg) is the product of cardiac output and arteriovenous oxygen difference [1], normalized to body mass. High CRF is independently associated with a lower risk for mortality and incident chronic diseases in general and clinical populations [2]. CRF can easily be improved by lifestyle changes (e.g., increase physical activity and reduce inactivity) and is directly modifiable for each individual. CRF provides a holistic overview of an individual's health status because it represents the interaction of the pulmonary, cardiovascular, and skeletal muscle systems. High CRF is associated with lower all‐cause mortality [3, 4], cardiovascular events [5], and other non‐communicable diseases like cancer and Type 2 diabetes mellitus (T2DM) [6]. The American Heart Association recommended implementing CRF as a vital sign. However, implementing cardiopulmonary exercise testing is complex and may not be available in all settings. Therefore, a metabolite‐based approach could serve as an efficient surrogate for CRF.

To gain insights into small molecules involved in energy production, a metabolomics approach may be used. This method allows measuring small molecules in the plasma, which may be produced by organs (e.g., the liver) or skeletal muscle. The relationship between CRF and the plasma metabolome is not fully understood. Previous studies reported that individuals with high CRF display a more favorable lipid profile since individuals with high CRF had lower low‐density lipoprotein (LDL) cholesterol, higher high‐density lipoprotein (HDL) cholesterol [7] and lower levels of acylcarnitine [8]. This observation may be explained by a better beta‐oxidation in persons with high CRF [9]. The underlying reason is increased mitochondrial density, greater enzyme activity, enhanced oxygen delivery, improved fat utilization, as well as faster recovery. Greater CRF is also related to lower levels of branched‐chain amino acids (BCAA) [10], which are elevated in individuals with insulin resistance and T2DM [11]. A previous study already developed a multi‐metabolite score to predict VO_2_peak [12]. Here, data from relatively healthy individuals (i.e., normal blood pressure and low incidence of T2DM) from the Framingham Heart Study was used to construct a “reduced score” of 61 metabolites, which was validated in the Coronary Artery Risk Development in Young Adults (CARDIA) study. Highlighting the importance of CRF for low chronic inflammation is supported by lower levels of C‐reactive protein (CRP), leptin, and insulin [13]. High chronic inflammation is related to a greater risk for cardiometabolic disease, which may be attenuated by high CRF [14, 15, 16]. Overall, previous research supports the systemic health benefits of high CRF.

The relationship between the plasma metabolome and CRF is different for men and women. For example, the concentration of BCAAs is higher in men, but glycine levels are higher in women [17]. The importance of sex is highlighted by the fact that equally high CRF is related to a lower mortality rates in women compared to men [18].

The aim of this study was to identify sex‐specific metabolic signatures related to CRF in two independent adult cohorts of the general population. Using two independent cohorts, including over 1600 individuals, enhances the probability of identifying relevant metabolites. An additional highlight of this analysis is that both cohorts underwent examination within the same time period, which ensures consistency. A better understanding of the sex‐specific metabolic signature of CRF may be useful to advance individualized medicine.

## 2 Materials and Methods

### 2.1 Study Population

The Study of Health in Pomerania (SHIP) is a population‐based research project. Men and women aged 20–79 years from the north‐east region of Germany were included in two independent study cohorts [19]. Background, sampling, and data collection details are described elsewhere [19]. Briefly, between 1997 and 2001, 6265 individuals were invited to participate in SHIP‐START‐0. In the baseline study (SHIP‐START‐0) 4308 (2193 women) individuals were included (68.8% response). The first follow‐up examination (SHIP‐START‐1) took place between 2002 and 2006 with a follow‐up response of 83.6% (*n* = 3300). Between 2008 and 2012, the second reexamination was conducted (SHIP‐START‐2) and 2333 subjects were included (follow‐up response 62.9%). Parallel to SHIP‐START‐2, a second independent cohort, SHIP‐TREND‐0, was recruited from the same region (*n* = 8826, aged 20–79 years). Individuals who already took part in SHIP‐START‐0 were excluded. For SHIP‐TREND‐0, 4420 subjects received a comprehensive healthcare screening (response 50.1%). All study participants gave written informed consent before taking part in the study. The study was approved by the ethics committee of the University of Greifswald and complies with the Declaration of Helsinki.

**FIGURE 1:**
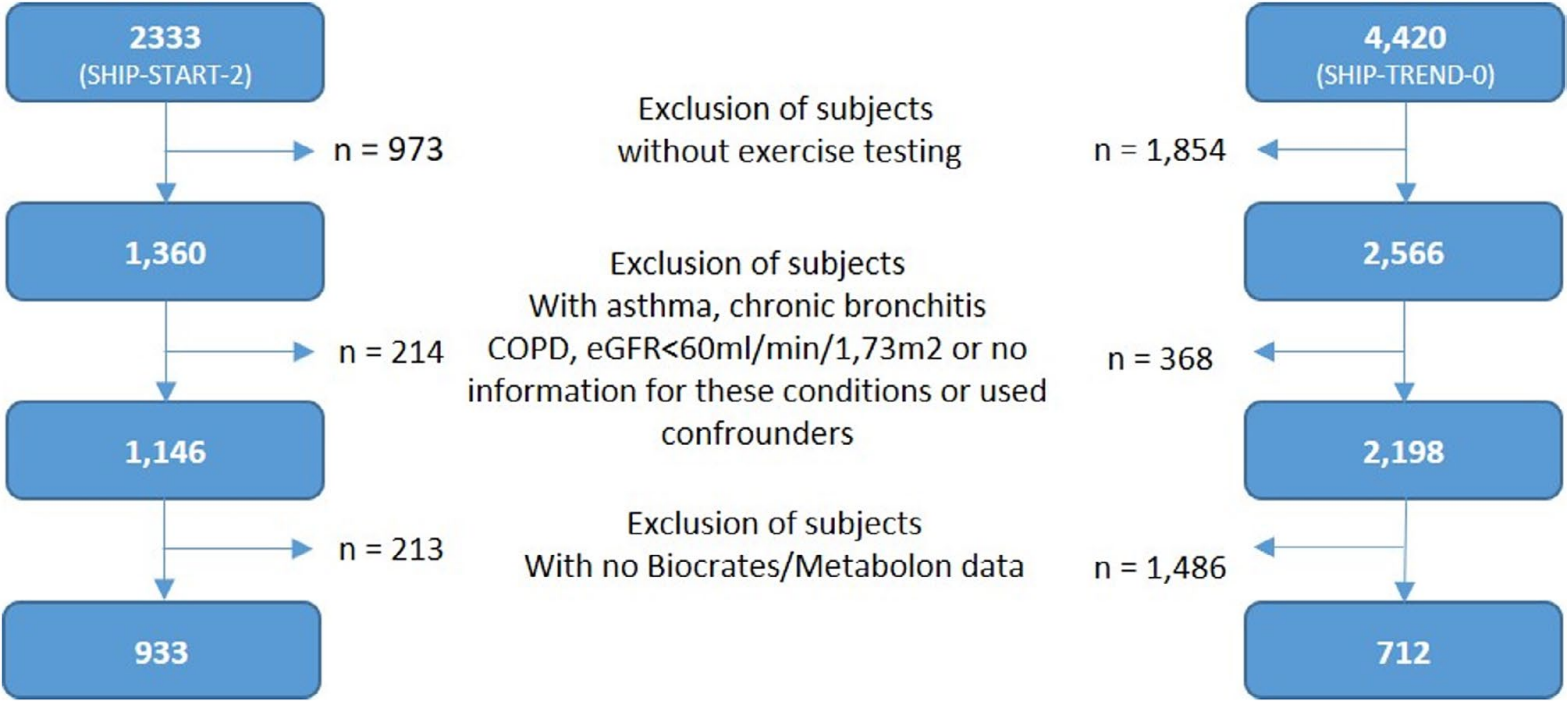
Flow chart of the study participants for SHIP‐START‐2 and SHIP‐TREND‐0.

Exercise testing data are available for 1360 SHIP‐START‐2 and 2566 SHIP‐TREND‐0 participants. From these subjects, we excluded individuals with at least one of the following conditions: asthma bronchial, chronic bronchitis, chronic obstructive pulmonary disease, estimated glomerular filtration rate (eGFR) < 60 mL/min/1.73m^2^, missing information for used confounders, and missing Biocrates or Metabolon (only SHIP‐TREND‐0) data. The final study populations included 933 SHIP‐START‐2 and 712 SHIP‐TREND‐0 participants (Figure 1).

### 2.2 Baseline Characteristics

In SHIP‐START‐2, data are available for 483 men with a median age of 58 years and 450 women with a median age of 56 years. In SHIP‐TREND‐0, information from 341 men and 371 women, both with a median age of 51 years, was used. In SHIP‐START‐2, the VO_2_peak median was 25.4 mL/min/kg in men (25th percentile: 21.2, 75th percentile: 30.7) and 21.4 mL/min/kg in women (18.2, 25.0). In SHIP‐TREND‐0, the median VO_2_peak was 29.3 mL/min/kg for men (24.5, 34.9) and 23.2 mL/min/kg for women (19.3, 27.1). Most of the participants were non‐ or ex‐smokers. The median BMI of all subgroups can be categorized as ‘Pre‐obesity’ according to the World Health Organization classification. Other baseline characteristics among the study participants are shown in Table 1.

**TABLE 1:** General characteristics of the study populations by sex.

	SHIP‐START‐2			SHIP‐TREND‐0		
	Men (*n* = 483)	Women (*n* = 450)	*p*	Men (*n* = 341)	Women (*n* = 371)	*p*
Age, years	58 (47; 68)	56 (46; 65)	0.02	51 (41; 61)	51 (41; 61)	0.74
Smoking, %						
Non‐smoker	28.6	51.1	< 0.01	32.2	49.9	< 0.01
Ex‐smoker	53.4	33.8		49.3	29.9	
Current smoker	18.0	15.1		18.5	20.2	
Alcohol consumption, g/day	10.0 (3.2; 21.4)	3.3 (1.3; 6.9)	< 0.01	8.3 (3.0; 17.4)	2.6 (0.7; 5.8)	< 0.01
Physical activity, %	71.6	75.1	0.24	72.7	74.7	0.61
Body mass, kg	87.1 (78.2; 96.4)	70.2 (62.1; 80.1)	< 0.01	86.5 (77.6; 96.2)	70.0 (62.2; 79.7)	< 0.01
Height, cm	176 (171; 180)	163 (159; 167)	< 0.01	177 (173; 180)	165 (160; 169)	< 0.01
BMI, kg/m^2^	28.0 (25.8; 30.7)	26.3 (23.8; 29.7)	< 0.01	27.8 (25.1; 30.3)	25.8 (23.1; 29.1)	< 0.01
Lean mass, kg	66 (61; 72)	47 (44; 50)	< 0.01	67 (61; 72)	47 (44; 51)	< 0.01
Fat mass, kg	21 (17; 25)	24 (18; 30)	< 0.01	20 (16; 25)	23 (18; 30)	< 0.01
Waist circumference, cm	97 (90; 104)	83 (76; 92)	< 0.01	94 (87; 102)	80 (74; 90)	< 0.01
Systolic blood pressure, mmHg	137 (127; 148)	126 (114; 138)	< 0.01	130 (120; 140)	118 (108; 129)	< 0.01
Hypertension, %	62.1	42.9	< 0.01	44.0	33.8	< 0.01
eGFR, mL/min/1.73m^2^	93 (81; 101)	92 (81; 102)	0.84	97 (88; 106)	97 (86; 107)	0.42
PeakVO_2_, mL/min/kg	25.5 (21.4; 31.1)	21.4 (18.1; 25.0)	< 0.01	29.3 (24.5; 34.9)	23.2 (19.3; 27.1)	< 0.01
VO_2_@AT, mL/min	1100 (950; 1300)	850 (750; 1000)	< 0.01	1200 (1000; 1400)	850 (750; 1000)	< 0.01
Maximum power output, watt	180 (148; 212)	132 (100; 148)	< 0.01	196 (164; 228)	132 (116; 148)	< 0.01

*Note:* Continuous data are expressed as median (Q1; Q3) nominal data are given as percentages.

Chi‐Quadrat test (nominal data) or Mann–Whitney test (interval data) were performed. BMI = body mass index, eGFR = estimated glomerular filtration rate.

### 2.3 Interview, Medical and Laboratory Examination

Certified and trained medical staff used standardized computer‐assisted interviews to ask the participants about age, sex, and smoking habits (current smoker, ex‐smoker, non‐smoker), physical activity behavior, and alcohol consumption. Physical inactivity was defined as less than 1 h per week of leisure time exercise, during winter or summer. Assessment of alcohol consumption (in grams of ethanol per day) was based on data regarding the consumption of beer, wine, and spirits during the last 30 days. History of exclusion criteria was self‐reported (chronic obstructive pulmonary disease, asthma bronchial and chronic bronchitis).

All participants underwent an extensive standardized medical examination. Anthropometric measurements were based on the recommendations of the World Health Organization and included height and body mass. Body mass was measured without shoes and in light clothing and documented to the nearest 0.1 kg using standard digital scales. Height measurement was performed by a calibrated scale. Body mass index (BMI) was calculated as body mass (kg) / height^2^ (m^2^). Waist circumference was measured between the lower rib margin and the iliac crest in a horizontal plane by using an inelastic tape and assessed to the nearest 0.1 cm. The subjects were standing comfortably with body mass evenly distributed between both feet. Fat‐free mass and fat mass were measured by bioelectrical impedance analysis using a multifrequency Nutriguard‐M device (Data Input, Pöcking, Germany) and the NUTRI4 software (Data Input, Pöcking, Germany) in participants without pacemakers.

Blood pressure was measured after a 5‐min resting period in a sitting position. Systolic and diastolic blood pressure were measured three times, with a 3‐min rest in between the measurements, on the right arm using a digital blood pressure monitor (HEM‐705CP, Omron Corporation, Tokyo, Japan). The mean of the second and the third readings was used for the present analyses. Hypertensive patients were defined by either self‐reported antihypertensive medication (defined as agents with anatomic, therapeutic and chemical code C02, C03, C07, C08, and C09) or a systolic blood pressure above 140 mmHg and/or diastolic values more than 90 mmHg.

Fasting (defined as at least 8 h since last meal) and non‐fasting venous blood samples were collected from all study participants in a supine position from the median cubital vein between 7 am and 4 pm. All samples were immediately analyzed or stored at −80°C in the Integrated Research Biobank (IRB) of University Medicine Greifswald and were used in accordance with the IRB regulations [20]. The eGFR was determined according to the Chronic Kidney Disease—Epidemiology Collaboration (CKD‐EPI) equation [21] based on creatinine and expressed in mL/min/1.73 m^2^.

### 2.4 Exercise Testing

CRF was measured by a symptom‐limited cardiopulmonary exercise test using a calibrated electromagnetically braked cycle ergometer (Ergoselect 100, Ergoline, Germany) according to a modified Jones protocol. After 3 min of rest, the participant underwent 1 min of unloaded cycling at 60 rpm (20 W), followed by a workload increase of 16 W/min until the test was ended by the participant due to exhaustion (RER > 1.05). All tests were performed at room air, with continuous monitoring of the electrocardiogram, blood pressure, and pulse oximetry. VO_2_peak was analyzed breath‐by‐breath over 10‐s intervals using a VIASYS HEALTHCARE system (Oxycon Pro, Rudolph's mask, JAEGER/VIASYS Healthcare system; Hoechberg, Germany) and was defined as the highest 10‐s average of absolute oxygen uptake during late exercise or early recovery [22].

### 2.5 Metabolomics Measurements

A detailed description of all applied measurement techniques is provided in the supplemental information. Briefly, in SHIP‐TREND‐0, untargeted metabolomics analysis for metabolic profiling was conducted at the Genome Analysis Center, Helmholtz Center Munich, Germany. Two separate LC–MS/MS analytical methods were used to obtain broad metabolite spectra in plasma samples in an untargeted manner. After preprocessing, 445 plasma metabolites remained for the statistical analyses. Some of the metabolite IDs could not be unambiguously assigned to the Metabolon chemical identity database and were referred to as “unknown metabolites” notated with “X” and a following unique number. In SHIP‐START‐2 and SHIP‐TREND‐0, targeted metabolomics profiling of the plasma samples was performed using the AbsoluteIDQ p180 Kit (BIOCRATES LifeSciences AG, Innsbruck, Austria). This approach allows simultaneous absolute quantification of up to 188 metabolites using liquid chromatography (Agilent 1260 Infinity Binary LC, Santa Clara, United States) and flow injection analysis–mass spectrometry (AB SCIEX 5500 QTrap mass spectrometer, AB SCIEX, Darmstadt, Germany).

### 2.6 Statistical Analysis

Categorical data were expressed as percentages; continuous data were expressed as median (Q1; Q3). For bivariate analyses, the Kruskal‐Wallis test (continuous data, log2‐transformed) or *χ* ^2^‐test (nominal data) were used to compare men and women. Multivariable linear regression models were performed to estimate the independent associations of VO_2_peak (exposure variable) with plasma metabolites (outcome variables) in men and women separately. The models were adjusted for age, height, and smoking. Analyses were separately performed in SHIP‐START‐2 and SHIP‐TREND‐0. To account for multiple testing, we adjusted the P values from regression analyses by controlling the false discovery rate (FDR) at 5% using the Benjamini‐Hochberg procedure. Statistical analyses were performed with SAS 9.4 (SAS Institute Inc., Cary, NC, USA).

Due to the use of both targeted and untargeted metabolic profiling in SHIP‐TREND‐0, some metabolites were identified using both methods. We decided to not include duplicates for our analysis. Metabolites identified using Metabolon were excluded if they were also part of the Biocrates p180 kit. Consequently, for men, nine metabolites and for women, five metabolites were excluded.

## 3 Results

### 3.1 Metabolites Related to CRF in Men and Women

**FIGURE 2:**
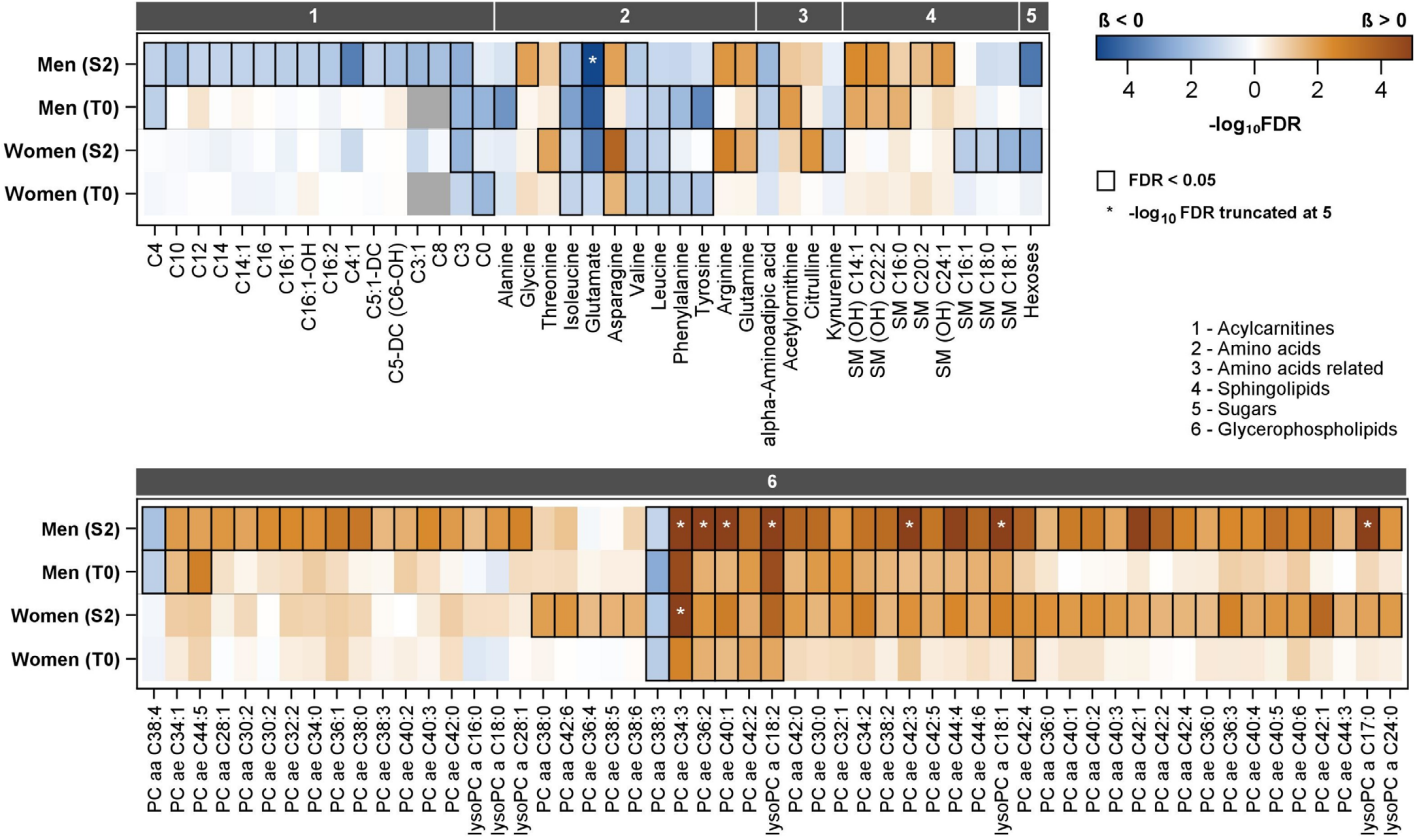
Color‐coded corrected P values (controlling the false discovery rate (FDR) at 0.05) for the association of plasma metabolites with PeakVO_2_ in men and women separately. Significant associations (FDR < 0.05) are marked with a black box. Linear regression models were adjusted for age, height and smoking status. Orange and blue shading indicate positive and inverse associations, respectively. Analyses were separately performed for SHIP‐START‐2 (S2) and SHIP‐TREND‐0 (T0). Metabolites were measured by targeted metabolomics (Biocrates). Gray indicates metabolites that were excluded in SHIP‐TREND‐0. Metabolites that were not significant in either men or women are only displayed in Figure S1.

**FIGURE 3:**
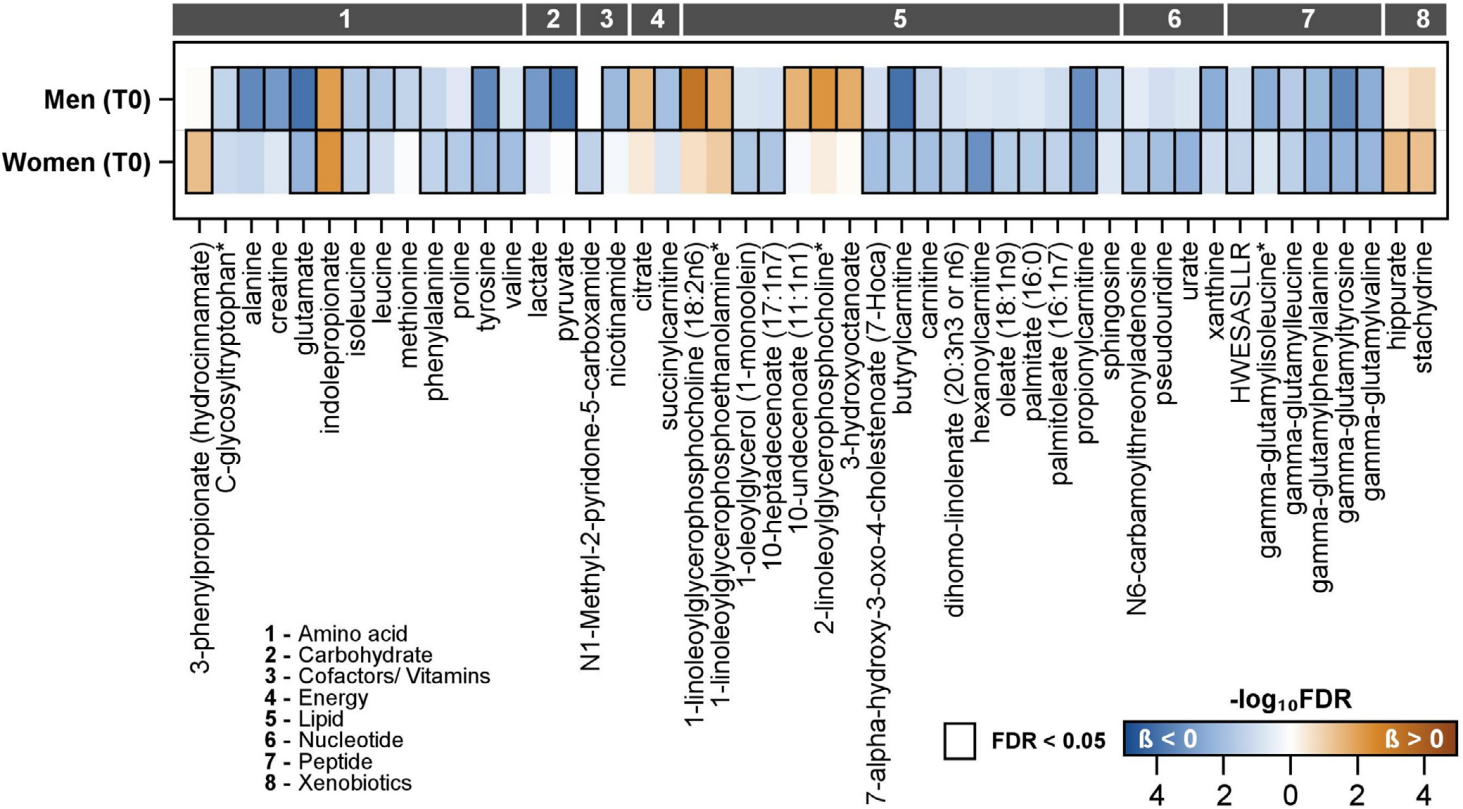
Color‐coded corrected *p* values (controlling the false discovery rate (FDR) at 0.05) for the association of plasma metabolites, measured by untargeted metabolomics (Metabolon), with PeakVO_2_ in SHIP‐TREND‐0 (T0) men and women separately. Significant associations (FDR < 0.05) are marked with a black box. Linear regression models were adjusted for age, height and smoking status. Orange and blue shading indicate positive and inverse associations, respectively. Unknown significant metabolites are only displayed in Figure S2.

The results for the targeted metabolomics analysis are presented in Figure 2 (Figure S1) while the untargeted metabolomics analysis is shown in Figure 3 (Figure S2). To better appreciate the similarities and differences between men and women, we have plotted the standardized coefficient estimates in SHIP‐START‐2 and SHIP‐TREND‐0 between men and women (Figure S3). Among all 132 metabolites significantly related to CRF, 41 were found for men and women in SHIP‐START‐2. Of these 41 metabolites, 35 were positively related to CRF. These included 32 lipids and three amino acids (asparagine, arginine and glutamine). The six metabolites with inverse relations to CRF independent of sex included two lipids (phosphatidylcholine aa C38:3 and propionylcarnitine), hexoses, as well as three amino acids (valine, isoleucine and glutamate).

Overall, 118 metabolites were related to CRF in SHIP‐TREND‐0. In men and women, 24 metabolites demonstrated positive associations with CRF, which included six lipids (five glycerophospholipids and indolepropionate) and an unannotated metabolite (X‐11315). A total of 17 metabolites (five amino acids, four lipids, four peptides and four metabolites with unknown function) were inversely related to CRF independent of sex.

### 3.2 Metabolites Divergent for Men and Women

Out of the 79 metabolites related to CRF in men in SHIP‐START‐2, 38 (22 positive, 16 inverse) were specific to men. Among those positively correlated with CRF, the majority are lipids (glycerophospholipids and sphingolipids), along with one amino acid (glycine). In contrast, inverse associations in men with CRF (*n* = 16) were almost exclusively related to acylcarnitines, followed by one glycerophospholipid and one amino acid‐related metabolite (alpha‐Aminoadaptic acid).

**FIGURE 4:**
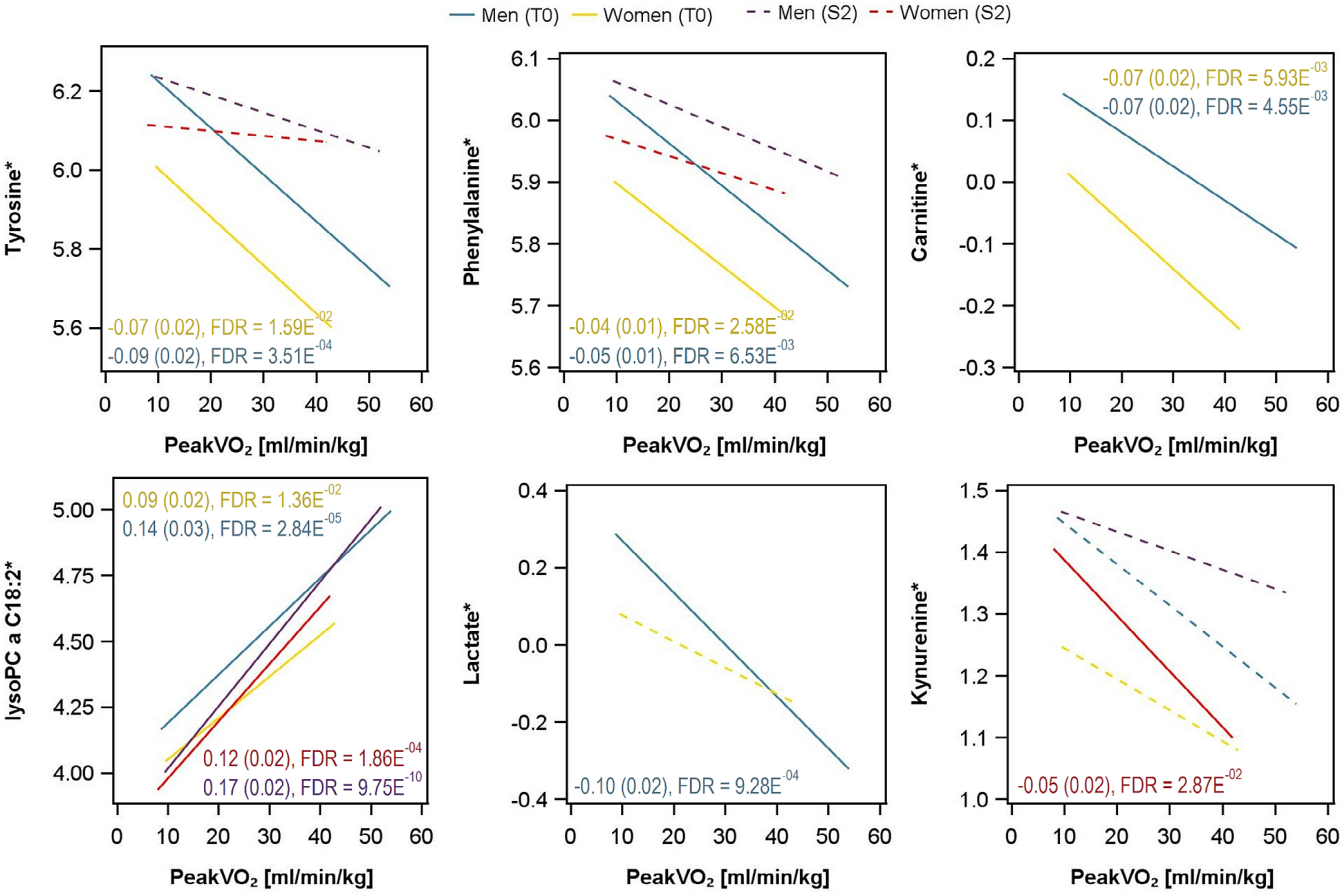
The linear association between tyrosine, phenylalanine, carnitine, lysoPCaC18:2, lactate, and kynurenine with peakVO_2_ (mL/min/kg) for men and women in SHIP‐START‐2 and SHIP‐ TREND‐0. *Metabolitelevels were log2‐transformed; beta estimated per standard deviation increase in peakVO_2_ with standard error were given. FDR = false discovery rate.

In women, a total of 53 metabolites were associated with CRF, with 12 (seven positive and five inverse) being unique to women in SHIP‐START‐2. Positive associations with CRF included five glycerophospholipids, citrulline, and threonine. Inversely associated with CRF in females were three sphingolipids, kynurenine (Figure 4) and leucine. Interestingly, the effect sizes were stronger in women, represented by an overall higher effect estimate.

In SHIP‐TREND‐0, of the 66 metabolites related to CRF in men, 42 were exclusively found in males (27 positive, 15 inverse). The majority of the positively associated metabolites were lipids (*n* = 20) including 15 glycerophospholipids, three sphingolipids, and two other lipids. Additionally, one amino acid (acetylornithine), one energy derivate (citrate) and five unidentified metabolites were positively related to CRF. The 15 metabolites with inverse associations included five amino acids, three lipids, two carbohydrates, one peptide, one cofactor (nicotinamide), one nucleotide (xanthine) and two unidentified metabolites.

We found 28 (six positive, 22 inverse) metabolites related to CRF uniquely in women. Positive associations with CRF in females included two amino acids, two xenobiotics (hippurate and stachydrine), one glycerophospholipid, and one unidentified metabolite. Inverse associations were found for 22 metabolites, comprising eight lipids, three nucleotides, two amino acids (valine and proline), one cofactor/vitamin, one peptide, and seven metabolites of unknown function. Similar to the observations in SHIP‐START‐2, stronger effect estimates were found in women compared to men in SHIP‐TREND‐0.

### 3.3 Metabolites Related to CRF in Women

Women in SHIP‐START‐2 showed 42 metabolites with positive relations to CRF. The majority of these metabolites were lipids, especially glycerophospholipids (*n* = 37), but also four amino acids (asparagine, arginine, threonine, glutamine) and one amino acid‐related metabolite (citrulline). Phosphatidylcholine aa C34:3 (β 0.053 95% confidence interval (CI) 0.034–0.072) had the strongest association with CRF. The largest group with inverse associations with CRF was lipids, including three sphingolipids, one glycerophospholipid (phosphatidylcholine aa C38:3) and one acylcarnitine (propionylcarnitine). Four amino acids (glutamate, valine, leucine and isoleucine) and kynurenine (Figure 4) were inversely related to CRF. The strongest inverse association was observed for glutamate (β −0.043 95% CI: −0.061 to −0.025) followed by hexoses (β −0.036 95% CI: −0.055 to −0.018).

In SHIP‐TREND‐0 52 metabolites (13 positive, 39 inverse) were related to CRF in women. Metabolites with positive associations included lipids (particularly glycerophospholipids), amino acids (indolepropionate, 3‐phenylpropionate and asparagine), xenobiotics (hippurate and stachydrin) and two metabolites with unknown function. The strongest positive association was identified for phosphatidylcholine ae C34:3 (β 0.046 95% CI: 0.026 to 0.067). The 39 metabolites with inverse relations to CRF included 12 lipids (including four acylcarnitines), seven amino acids, five peptides, three BCAAs (leucine, valine and isoleucine), three nucleotides (urate, pseudouridine and N6‐carbamoylthreonyladenosine), N1‐Methyl‐2‐pyridone‐5‐carboxamide, and 11 metabolites with unknown function. The acylcarnitine hexanoylcarnitine displayed the strongest inverse association (β −0.046 95% CI: 0.065 to −0.027).

### 3.4 Metabolites Related to CRF in Men

In men, 79 metabolites (57 positive, 22 inverse) were related to CRF in SHIP‐TREND‐0. Among the positive associations, 53 were lipids, including 49 glycerophospholipids and four sphingolipids. The strongest association was found for lysophosphatidylcholine a C18:2 (β 0.05 95% CI: 0.036 to 0.064) and hydroxysphingomyelin C14:1 (β 0.026 95% CI: 0.011 to 0.041). Additionally, the four amino acids arginine, asparagine, glycine, and glutamine showed positive associations with CRF. The metabolites with inverse associations included 15 acylcarnitines, two glycerophospholipids, three amino acids (glutamate, isoleucine and valine), hexose (including glucose) and alpha‐aminoadipic acid. Within the acylcarnitines, butenylcarnitine (β −0.033 95% CI: −0.048 to −0.018) and within the glycerophospholipids, phosphatidylcholine aa C38:4 (β −0.022 95% CI: 0.037 to −0.007) had the largest effect estimates. Among all inverse associations, glutamate showed the strongest inverse relationship with CRF (β −0.048 95% CI: −0.063 to −0.034).

In SHIP‐TREND‐0, men showed a total of 66 metabolites related to CRF (34 positive, 32 inverse). Most of the positive associations were observed in lipids (including 20 glycerophospholipids, three sphingolipids, 3‐hydroxyoctanoate and 10‐undecenoate (11:1n1)), one amino acid (indolepropionate), acetylornithine, one energy derivate (citrate) and six unidentified metabolites. The strongest positive association was identified in phosphatidylcholine ae C34:3 (β 0.045 95% CI: 0.029 to 0.062). Overall, 32 metabolites were inversely related to CRF. These included ten amino acids or amino acid‐related metabolites, seven lipids (four acylcarnitines, two glycerophospholipids and sphingosine), five peptides, and two carbohydrates (pyruvate and lactate). Pyruvate (β −0.044 95% CI: −0.061 to −0.027) had the strongest inverse association with CRF, followed by glutamate (β −0.043 95% CI: −0.059 to −0.026). The nucleotide xanthine and nicotinamide were also related to CRF. Six metabolites with unknown function also had inverse associations with CRF. Among these, the metabolite X‐12096 showed the strongest inverse association with CRF (β −0.047 95% CI: −0.065 to −0.028).

## 4 Discussion

High CRF is associated with multiple positive health outcomes. Metabolic profiling may help to improve our understanding of the underlying biology related to the health benefits of high CRF. We identified metabolic signatures of CRF in two independent population‐based cohorts. Most metabolites related to CRF in our analysis were lipids and amino acids (Figures 2 and 3). We also identified inverse associations for acylcarnitines with CRF (Figure 4). Given that acylcarnitines are associated with poor metabolic health, this may provide a mechanistic link to the adverse health effects of low CRF [23, 24]. The majority of the glycerophospholipids showed positive associations with CRF. Interestingly, sphingolipids showed positive and inverse relations with CRF [3]. BCAAs were inversely associated with CRF in both cohorts. Other amino acids, like arginine, were elevated when CRF was higher. Importantly, we also found sex‐specific metabolic signatures for CRF, with women having much stronger effect estimates compared to men.

### 4.1 Lipids and Lipid Like Molecules

The metabolic signature of CRF was largely influenced by lipids. There was no clear direction for the relationship between lipids and CRF, as reported previously [12]. The majority of the sphingolipids displayed positive relations to CRF. This agrees with the results of a study showing an accumulation of sphingolipids after physical exercise [25]. This is particularly interesting given that sphingolipids are considered to be drivers of cardiovascular disease [26]. The metabolite lysoPC a C18:2 had positive associations with CRF in men and women in both cohorts (Figure 4). This metabolite is related to a longer lifespan [27]. Moreover, phosphatidylcholines that are highly polyunsaturated seem to be inversely associated with total mortality [27]. Previously, lysoPC a 18:1and lysoPC a 18:2 were inversely associated with waist circumference, whereas PC aa C38:3 and PC aa C38:4 showed positive associations [28]. Hence, our findings support the notion that a higher CRF is related to a lower waist circumference. In obese individuals with and without T2DM, lysoPC levels are reduced [29]. Similarly, persons with impaired glucose tolerance also have lower levels of lysoPC [30]. In summary, previous studies reported lower levels of lysoPC in the presence of metabolic disorders. Since a higher CRF was associated with greater lysoPC levels in both cohorts and both sexes (Figure 4), one may speculate that higher lysoPC levels in fitter individuals may be part of the explanation for the health‐promoting metabolic effect of high CRF.

Mitochondrial capacity (in skeletal muscle) is impaired in individuals with insulin resistance and T2DM [31]. Therefore, a higher concentration of acylcarnitines in individuals with low CRF may reflect impaired mitochondrial function for fatty acid oxidation [32]. Moreover, similar to lysoPC, acylcarnitines are increased in individuals with obesity and T2DM [23, 24]. Lower acylcarnitines are associated with a progression from normoglycemia to prediabetes [33]. Recently, acylcarnitines were associated with hypertension [34] and coronary artery disease [35]. A review summarized that acylcarnitines are not only involved in insulin resistance and T2DM, but also influence cardiac function, pro‐inflammatory processes, and lipotoxicity [36]. Hence, higher acylcarnitines may be on mechanisms that link low CRF to poor metabolic health outcomes.

### 4.2 Amino Acids and Amino Acids Related

Higher in persons with obesity [37], BCAA levels are associated with cardiovascular and metabolic risk factors, regardless of BMI [38]. Higher BCAA concentrations are also related to mitochondrial dysfunction leading to impaired fatty acid and glucose oxidation [39]. We found lower BCAAs in study participants with higher CRF. Elevated BCAAs are associated with lower VO_2_peak, and physical exercise decreases plasma BCAA concentrations [25, 40]. This supports the notion that individuals with a higher CRF also have a better mitochondrial capacity, as well as a lower risk for cardiometabolic disease mediated by lower BCAAs.

We identified a positive association between arginine and CRF. This vasodilator promotes endothelial function as a precursor of nitric oxide [41]. Whether the observed association is truly related to CRF and not to dietary habits cannot be concluded based on our results since we are unable to adjust for diet. A recent study developed a metabolic fitness score which includes arginine supports our finding [12]. Besides arginine, several other amino acids (e.g., alanine, phenylalanine and tyrosine; Figure 4) showed inverse associations with CRF. These observations agree with a reduction of phenylalanine (Figure 4) and tyrosine after cardiopulmonary exercise testing [40]. Given that these amino acids are associated with metabolic risk factors like systolic blood pressure and BMI, one may speculate that the biological signaling mechanisms of a high CRF are mediated through these amino acids.

### 4.3 Sex‐Specific Metabolic Profiles

Our data revealed that some metabolites were associated with CRF independent of sex, whereas other metabolites were only related to CRF in either women or men. A previous study already showed that sex has a significant effect on more than 100 metabolites [42]. Interestingly, associations with acylcarnitines were almost exclusively related to CRF in men. Acylcarnitine concentrations are also higher in men compared to women [42]. Men also have significantly greater levels of circulating BCAAs [43, 44]. We show that in women the effect estimates for the association between amino acids and CRF were higher compared to men. Discrete differences of metabolites between men and women after exercising have been reported before, with women showing a greater reduction of kynurenine and men showing higher levels of lactate [40]. Hence, our results suggest that the health‐promoting effect of a higher CRF could be greater for women. Accordingly, women can achieve a greater reduction in all‐cause and cardiovascular mortality from physical activity compared to men [45].

An additional interesting sex‐specific outcome was the observed opposite association between sphingolipids, specifically sphingomyelins, and CRF. While positive associations were found in men, women had inverse relations. Elevated levels of sphingomyelins are associated with an increased risk for coronary artery disease [46]. Such a directional inconsistency between both sexes could not be shown previously in associations between proteomics and CRF [13]. However, more research is necessary to uncover the complex relationship between sex, CRF, and cardiovascular outcomes/risk factors.

### 4.4 Review of Recent Literature

Our finding aligns with prior research examining metabolic signatures for CRF and metabolic responses to exercise [25, 40]. Non‐directional associations with several lipid metabolites have been identified, while elevated BCAA levels were linked to lower VO_2_peak. Although previous studies have reported minor sex‐specific differences [40], our analysis reveals contrasting associations in sphingolipid levels between both sexes. Using data from the Framingham Heart Study, a metabolic score for CRF has been developed to predict VO_2_peak, which is in agreement with some of our metabolites (lysoPC C24:0) but also shows opposite associations with CRF compared to our results (e.g., alanine) [12]. While previous research focused mostly on healthy individuals, our sample includes a large number of participants with prevalent subclinical cardiometabolic diseases. Further research is necessary to elucidate the biological mechanisms of CRF and acute exercise.

### 4.5 Strengths and Limitations

Some limitations of our study should be mentioned. First, the cross‐sectional study design does not allow any statements about causality. Furthermore, factors that influence the plasma metabolome, such as medication or diet, were not included in our model. As SHIP is based in north‐east Germany with mostly Caucasians, there is only a limited transferability of our results to other ethnicities. This study also has several strengths; we included more than 1600 individuals from two independent population‐based cohorts. Another strength of SHIP is broad health screening without a focus on specific diseases. Moreover, the combination of targeted and untargeted metabolic profiling increased the likelihood of identifying metabolites related to CRF.

## 5 Perspective

Many circulating plasma metabolites are associated with CRF. We identified several metabolites known for their roles with regard to poor metabolic health (e.g., BCAAs and acylcarnitines) which were inversely associated with CRF. The majority of the identified metabolites were lipids, especially glycerophospholipids, followed by amino acids. These easily accessible plasma metabolites could be used to predict an individual's cardiometabolic disease risk. We also found sex‐related metabolites and a stronger association between the metabolites and CRF in women. The sexual dimorphism and related factors (hormonal differences, body composition and different dietary patters) might be part of the explanation, but more research is necessary to understand the biological mechanisms associated with the health benefits of higher CRF.

## Conflicts of Interest

The authors declare no conflicts of interest.

## Supporting information


Data S1.

## Data Availability

Data from the “Study of Health of Pomerania” are available from the University Medicine Greifswald, Germany but restrictions apply to the availability of these data. Data are, however, available upon reasonable request at https://transfer.ship‐med.uni‐greifswald.de/FAIRequest/data‐use‐intro and with permission of the University Medicine Greifswald.
