# Supplementary material for: The Metabolic Signature of Cardiorespiratory Fitness
Source: Scand J Med Sci Sports. 2025 Mar 12;35(3):e70034. doi: 10.1111/sms.70034 (PMC11899505; doi:10.1111/sms.70034)
Supplement: Supplementary file 1 — Data S1. [file SMS-35-e70034-s001.docx]

**SUPPLEMENTAL INFORMATION**

**The metabolic signature of cardiorespiratory fitness**

Julia Bork^1,2^, Marcello RP Markus^1,2,3^, Ralf Ewert^1^, Matthias Nauck^2,4^, Christian Templin^1,2^, Henry Völzke^2,5^, Gabi Kastenmüller^6^, Jerzy Adamski^8,9,10^, Anna Artati^7^, Marcus Dörr^1,2^, Nele Friedrich^2,4^, Martin Bahls^1,2^

**FIGURES**


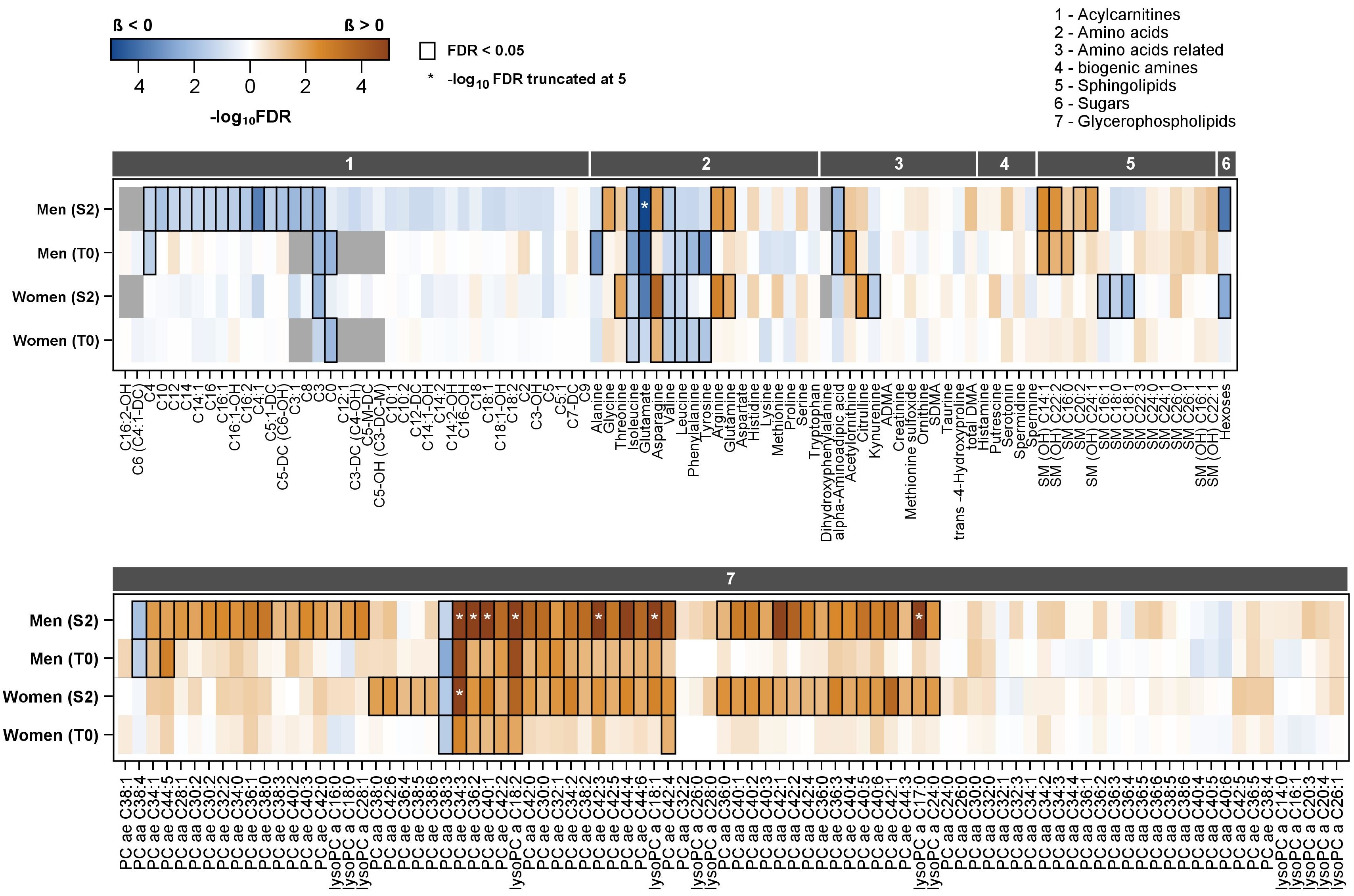


**Figure S1**. Color-coded corrected P values (controlling the false discovery rate (FDR) at 0.05) for the association of plasma metabolites with PeakVO_2_ in men and women separately. Significant associations (FDR < 0.05) are marked with a black box. Linear regression models were adjusted for age, height and smoking status. Orange and blue shading indicate positive and inverse associations, respectively. Analyses were separately performed for SHIP-START-2 (S2) and SHIP-TREND-0 (T0). Metabolites were measured by targeted metabolomics. Grey indicates metabolites that were excluded in SHIP-TREND-0.


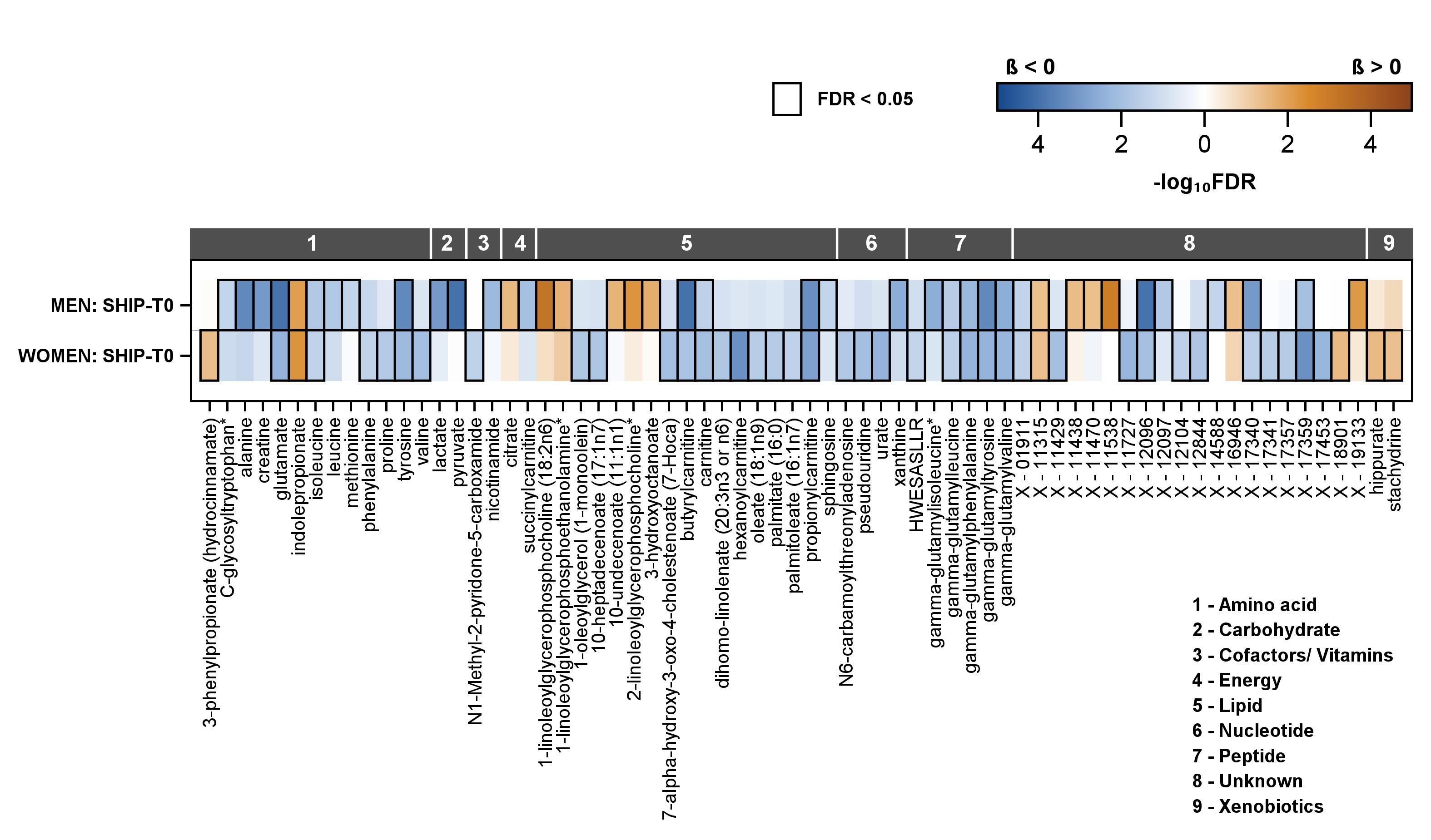


**Figure S2**. Color-coded corrected P values (controlling the false discovery rate (FDR) at 0.05) for the association of plasma metabolites, measured by untargeted metabolomics, with PeakVO_2_ in SHIP-TREND-0 (T0) men and women separately. Significant associations (FDR < 0.05) are marked with a black box. Linear regression models were adjusted for age, height and smoking status. Orange and blue shading indicate positive and inverse associations, respectively. Unknown

metabolites are marked with X-.


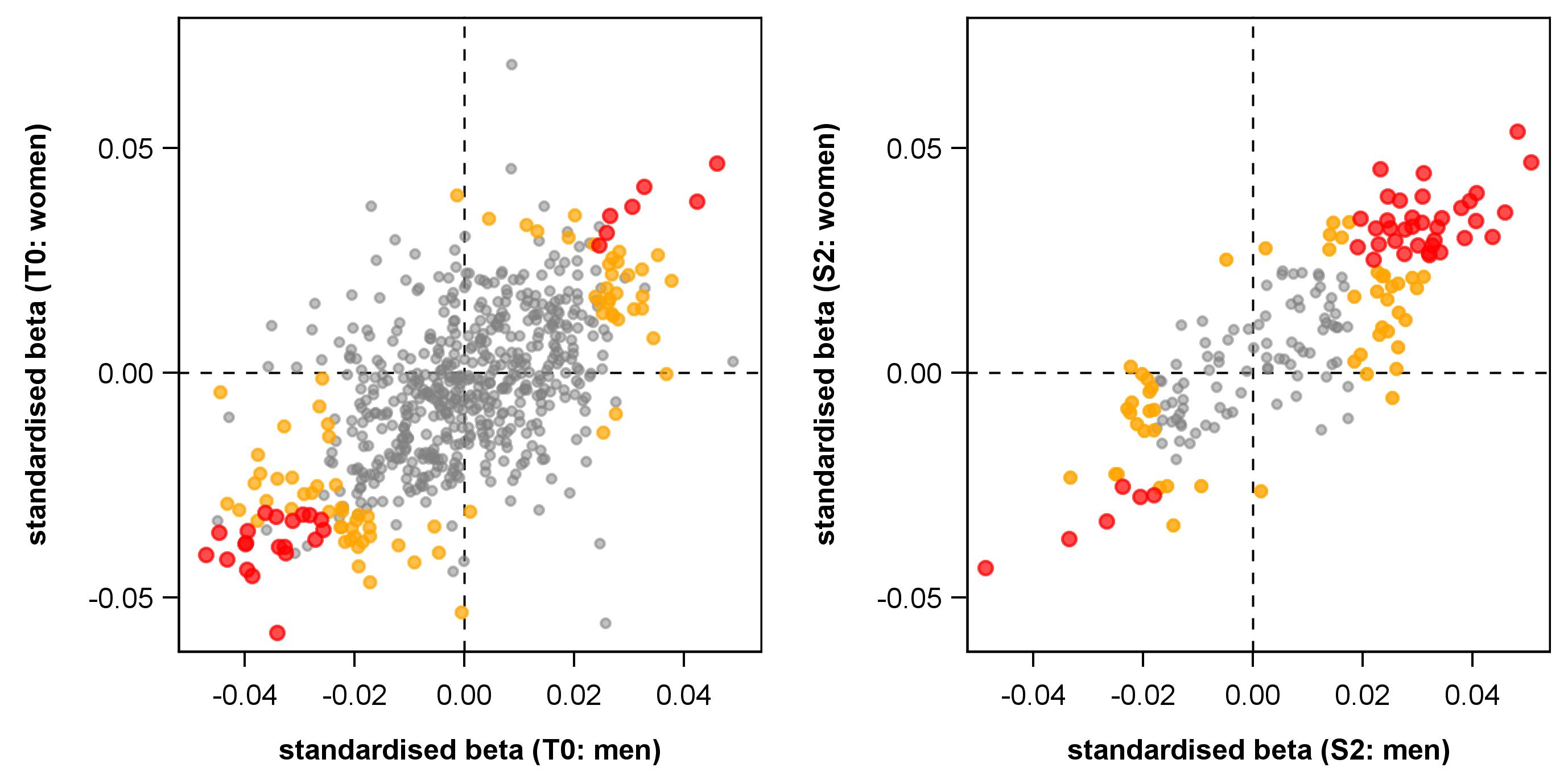


**Figure S3.** Comparison of standardised beta coefficient calculated by linear regression models (see figure S1 and S2) between men and women separately in SHIP-START-2 (S2, right side) and SHIP-TREND-0 (T0, left side). Red circles represent metabolites that were significant in both men and women. Orange circles represent metabolites that were either significant in men or women.

**METHODS**

***Untargeted LC-MS/MS profiling***

*Metabolomics measurements*

Untargeted metabolomics analysis for metabolic profiling was conducted at the Genome Analysis Center, Helmholtz Center Munich. Two separate LC-MS/MS analytical methods as previously described, i.e. in positive and in negative electrospray ionization modes, were used to detect a broad metabolite panel [1]. In this study, samples were divided into two sets according to the biological matrices of the samples, i.e. plasma. On the day of extraction, samples were thawed on ice. Of each sample 100 µL were pipetted into a 96-well plate (2 mL volume per well). In addition to study samples, a pooled human reference plasma sample (Seralab, West Sussex, United Kingdom) and another pooled reference matrix of each sample set (Seralab, West Sussex, United Kingdom) were extracted (1 well and 6 wells, respectively). These samples served as technical replicates throughout the data set to assess process variability. Beside those samples, 100 μL of water was extracted as samples to serve as process blanks (6 wells). Protein was precipitated and the metabolites were extracted with 475 µL methanol, containing four recovery standards to monitor the extraction efficiency. After centrifugation, the supernatant was split into 4 aliquots of 100 µL each onto two 96-well microplates. The first 2 aliquots were used for LC-MS/MS analysis in positive and negative electrospray ionization mode. Two further aliquots were kept as a reserve. The extracts were dried on a TurboVap 96 (Zymark, Sotax, Lörrach, Germany). Prior to LC-MS/MS in positive ion mode, the samples were reconstituted with solvents compatible with each of both ionization methods (50 µL of plasma). Reconstitution solvents for both ionization modes contained internal standards that allowed monitoring of instrument performance and also served as retention reference markers. To minimize human error, liquid handling was performed by a Hamilton Microlab STAR robot (Hamilton Bonaduz AG, Bonaduz, Switzerland). LC-MS/MS analysis was performed on a linear ion trap LTQ XL mass spectrometer (Thermo Fisher Scientific GmbH, Dreieich, Germany) coupled with a Waters Acquity UPLC system (Waters GmbH, Eschborn, Germany). Two separate columns (2.1 x 100 mm Waters BEH C18, 1.7 µm particle-size) were used either for acidic (solvent A: 0.1% formic acid in water, solvent B: 0.1% formic acid in methanol) and or for basic (A: 6.5 mM ammonium bicarbonate, pH 8.0, B: 6.5 mM ammonium bicarbonate in 95% methanol) mobile phase conditions, optimized for positive and negative electrospray ionization, respectively. After injection of the sample extracts, the columns were developed in a gradient of 99.5% A to 98% B over an 11 min run time at 350 µL/min flow rate. The eluent flow was directly run through the ESI source of the LTQ XL mass spectrometer. The mass spectrometer analysis alternated between MS and data-dependent MS/MS scans using dynamic exclusion and the scan range was from 80-1000 m/z. Metabolites were identified by Metabolon, Inc. from the LC-MS/MS data by automated multiparametric comparison with a proprietary library, containing retention times, m/z ratios, and related adduct/fragment spectra [2]. Identification criteria for the detected metabolites are described in Evans *et al.* [1].

*Metabolomics measurements: quality control and normalization of metabolite levels*

To correct for daily variations of platform performance, the raw ion count of each metabolite was rescaled by the respective median value of the run day. Valid estimation of the median was ensured by keeping only metabolites with at least three measured values on more than the half of the run days. This procedure resulted in 738 metabolites for plasma available for the present analysis. All metabolite levels were log_2_-transformed. We performed multivariate outlier detection using an algorithm proposed by Filzmoser *et al.* [3] as implemented in the *pcout* function within the R package *mvoutlier*. The algorithm provides an outlier score for each sample based on a weighted combination of location and scatter estimations using principal component analysis and the Mahalanobis distance on a robustly scaled data matrix. The default parameters were used for the identification process, except the critical value for the location outliers was set to 4, as it corresponds to a 4 SD exclusion criteria. The minimum score was used as cut-off for outlier identification. As a result, 13 plasma samples were excluded.

***Targeted LC-MS/MS profiling***

*Metabolomics measurements*

Targeted metabolomics profiling of the plasma samples was performed using the AbsoluteIDQ p180 Kit (BIOCRATES LifeSciences AG, Innsbruck, Austria, online supplementary methods). 10 µL aliquots of each plasma sample were processed as recommended by the manufacturer. The fully automated assay combined flow injection (FIA) and LC-MS/MS selective detection using MRM pairs and quantifies up to 188 metabolites from 5 different compound classes. Via FIA acyl carnitines, phospho- and sphingolipids were measured in positive ionization mode and the sum of hexoses in negative ionization mode. With a LC-MS/MS analytical method, under the use of an Agilent C18 column, amino acids and biogenic amines were detected. MS analyses were performed on an AB SCIEX 5500 QTrap™ mass spectrometer (AB SCIEX, Darmstadt, Germany) with electrospray ionization combined with a HPLC system (Agilent 1260 Infinity Binary LC, Santa Clara, United States) including a degasser unit, column oven, autosampler and a binary pump. Internal standards (isotope labelled) are partially integrated in the Kit plate for metabolite quantification. After the measurement a pre-processing step, includes peak integration and concentration determination from calibration curves, with Analyst software (Version 1.5.1, AB Sciex, Darmstadt, Germany), data were uploaded into Biocrates MetIDQ software (part of the kit) and the metabolite concentrations were automatically calculated with it.

*Metabolomics measurements: quality control and normalization of metabolite levels*

To account for between-plate variation, a sole sample dependent normalization was performed. To this end for each plate the measured concentrations of the metabolites were divided by the median concentration leading to equal median values for each metabolite on each plate. Subsequently, the median of the plate medians was calculated to reset to the original scale (µM concentrations). No obvious pattern in missing values along the measurement period became obvious. However, only metabolites with at least 20% valid observations were included in the final data sets, resulting in 183 used for subsequent analysis. PCA was performed to detect multivariate outliers. These were defined as samples deviating more than three times the standard deviation (SD) from the mean Mahalanobis distance based on the first ten principle components. As a result, four samples were excluded. Finally, metabolite levels were log_2_-transformed.

**References**

[1] A.M. Evans, C.D. DeHaven, T. Barrett, M. Mitchell, E. Milgram, Integrated, nontargeted ultrahigh performance liquid chromatography/electrospray ionization tandem mass spectrometry platform for the identification and relative quantification of the small-molecule complement of biological systems, Analytical chemistry 81(16) (2009) 6656-67.

[2] K.A. Lawton, A. Berger, M. Mitchell, K.E. Milgram, A.M. Evans, L. Guo, R.W. Hanson, S.C. Kalhan, J.A. Ryals, M.V. Milburn, Analysis of the adult human plasma metabolome, Pharmacogenomics 9(4) (2008) 383-97.

[3] Filzmoser, Peter, Ricardo Maronna, and Mark Werner. "Outlier identification in high dimensions." Computational statistics & data analysis 52.3 (2008): 1694-1711.
